# Aortic Stiffness in Lone Atrial Fibrillation: A Novel Risk Factor for Arrhythmia Recurrence

**DOI:** 10.1371/journal.pone.0076776

**Published:** 2013-10-03

**Authors:** Dennis H. Lau, Melissa E. Middeldorp, Anthony G. Brooks, Anand N. Ganesan, Kurt C. Roberts-Thomson, Martin K. Stiles, Darryl P. Leong, Hany S. Abed, Han S. Lim, Christopher X. Wong, Scott R. Willoughby, Glenn D. Young, Jonathan M. Kalman, Walter P. Abhayaratna, Prashanthan Sanders

**Affiliations:** 1 Centre for Heart Rhythm Disorders, University of Adelaide and Royal Adelaide Hospital, Adelaide, Australia; 2 Department of Cardiology, Royal Melbourne Hospital and the Department of Medicine, University of Melbourne, Victoria, Australia; 3 Academic Unit of Internal Medicine, Canberra Hospital, Canberra, Australia; Emory University, United States of America

## Abstract

**Background:**

Recent community-based research has linked aortic stiffness to the development of atrial fibrillation. We posit that aortic stiffness contributes to adverse atrial remodeling leading to the persistence of atrial fibrillation following catheter ablation in lone atrial fibrillation patients, despite the absence of apparent structural heart disease. Here, we aim to evaluate aortic stiffness in lone atrial fibrillation patients and determine its association with arrhythmia recurrence following radio-frequency catheter ablation.

**Methods:**

We studied 68 consecutive lone atrial fibrillation patients who underwent catheter ablation procedure for atrial fibrillation and 50 healthy age- and sex-matched community controls. We performed radial artery applanation tonometry to obtain central measures of aortic stiffness: pulse pressure, augmentation pressure and augmentation index. Following ablation, arrhythmia recurrence was monitored at months 3, 6, 9, 12 and 6 monthly thereafter.

**Results:**

Compared to healthy controls, lone atrial fibrillation patients had significantly elevated peripheral pulse pressure, central pulse pressure, augmentation pressure and larger left atrial dimensions (all P<0.05). During a mean follow-up of 2.9±1.4 years, 38 of the 68 lone atrial fibrillation patients had atrial fibrillation recurrence after initial catheter ablation procedure. Neither blood pressure nor aortic stiffness indices differed between patients with and without atrial fibrillation recurrence. However, patients with highest levels (≥75^th^ percentile) of peripheral pulse pressure, central pulse pressure and augmentation pressure had higher atrial fibrillation recurrence rates (all P<0.05). Only central aortic stiffness indices were associated with lower survival free from atrial fibrillation using Kaplan-Meier analysis.

**Conclusion:**

Aortic stiffness is an important risk factor in patients with lone atrial fibrillation and contributes to higher atrial fibrillation recurrence following catheter ablation procedure.

## Introduction

Atrial fibrillation (AF) has been recognized as an emerging epidemic. Increased focus on its prevention is thereby warranted given the limitations with current therapeutic options [1]. Recently, aortic stiffness has been proposed as a novel modifiable risk marker for AF, with increased brachial pulse pressure found to be associated with AF development in the Framingham community-based observational cohort [2]. Importantly, this association was stronger than systolic blood pressure alone and remained significant even after adjustment for established predictive factors such as left atrial enlargement and left ventricular hypertrophy [2].

However, peripheral derived blood pressures are known to overestimate true central hemodynamic indices [3]. In particular, central pressures are important measures of subclinical organ damage with greater patho-physiological relevance than peripheral pressures and therefore, better at predicting disease progression and outcomes, including coronary restenosis and cardiovascular mortality [3-5]. Central pulse wave analysis is a clinically validated, non-invasive and reproducible method for assessment of aortic stiffness [5-7]. Radial artery waveform obtained by applanation tonometry can be used to derive the following surrogate measures of aortic stiffness: central systolic blood pressure, central pulse pressure, augmentation pressure and augmentation index. In individuals with aortic stiffness, increased pulse wave velocity results in merging of the incident and reflected arterial waves leading to increased augmentation pressure as well as central systolic and pulse pressures.

In this study, we aim to further investigate the role of aortic stiffness in AF by analyzing the central pulse wave of lone AF patients who have no apparent structural heart disease or conditions that predispose them to the arrhythmia. Recently, it has been demonstrated that lone AF patients have an abnormal atrial electrical and structural substrate [8-10]. We posit that aortic stiffness contributes to adverse atrial remodeling that result in the development and persistence of lone AF. The impact of such a consequence was studied by examining arrhythmia recurrence following catheter ablation in patients with lone AF.

## Methods

### Study Population

The Research Ethics Committee of the Royal Adelaide Hospital, Australia approved this study. From a total of 115 consecutive lone AF patients who underwent initial catheter ablation procedure at our institution for symptomatic AF between August 2005 and September 2010, 72 agreed to participate in this study. Due to the presence of AF during applanation tonometry in 4 patients, we only included data from the remaining 68 patients for analysis. Lone AF was defined as previously described by: absence of structural heart disease or stroke based on history, physical examination, chest X-ray, routine blood chemistry, and echocardiography; coronary artery disease was excluded by clinical, ECG or stress test criteria; pulmonary disease, hypertension, hyperthyroidism and diabetes were eliminated by appropriate tests [11]. An additional 50 age- and sex-matched control subjects with no echocardiographic evidence of structural heart disease or risk factors for AF were also recruited from the local community. All patients and control subjects gave informed written consent to the study protocol.

### Blood Pressure and Radial Artery Pulse Wave Measurements

Measurements of blood pressure and radial artery applanation tonometry were performed in a quiet, temperature controlled room (22°C) with subjects in a recumbent position after 10 minutes of rest. All subjects were instructed to refrain from alcohol and caffeine consumption for 1 day prior to these measurements. Brachial blood pressure was the average of the last 2 readings (out of 3) measured in each subject at 5-minutes interval using a mercury sphygmomanometer and appropriately sized arm cuff.

**Figure 1:**
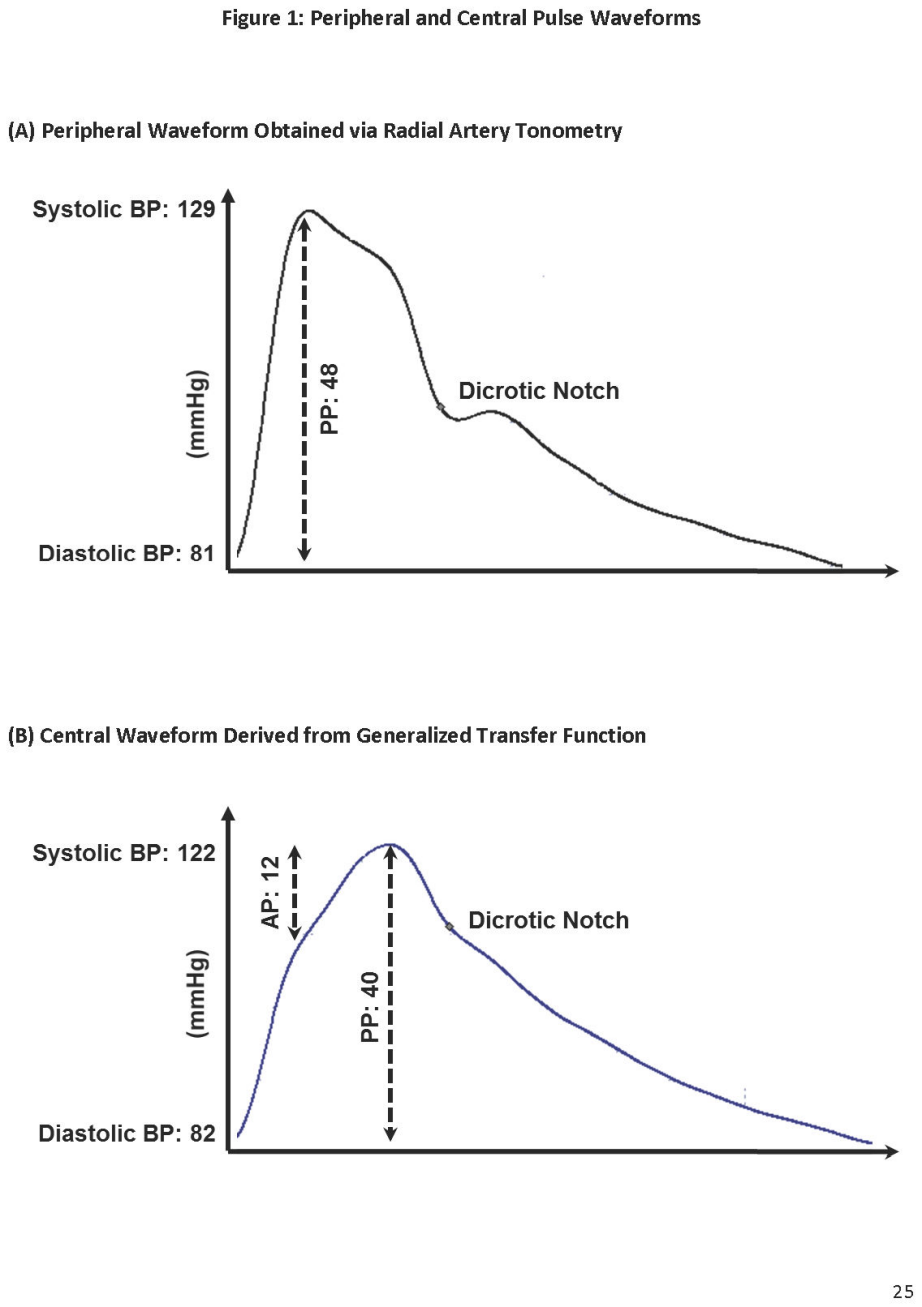
Peripheral and Central Pulse Waveforms. (A) This figure depicts a peripheral waveform obtained via radial artery tonometry. In this sample from a lone AF patient, peripheral blood pressure is 129/81 with a pulse pressure of 48 mmHg. (B) Aortic waveform can then be derived from peripheral waveform shown in (A) with the SphygmoCor software’s generalized transfer function. The central blood pressure is 122/82 with a pulse pressure of 40 mmHg. Augmentation pressure is a measure of reflected pressure wave, which is the difference between aortic systolic pressure and the first peak of the waveform. Augmentation index in this example is 12/40 X 100 = 30%.

Radial artery pressure waveform was acquired using a high fidelity applanation tonometer on the same arm (SphygmoCor Version 9, Atcor Medical, Itasca, IL, USA). The system has real-time monitoring of waveform quality (Figure 1A) allowing automatic capture once 11 seconds of acceptable data is obtained. The algorithm monitored each recording for pulse height, diastolic and shape variation. Quality of acquired data was also verified manually to ensure inclusion of only high quality recordings with operator index of >90%. Aortic pressure waveform was then derived using a validated generalized transfer equation [12]. Three measurements were performed on each subject to provide a mean value for each parameter: Central and peripheral pulse pressure – taken as the difference between their respective systolic and diastolic readings; Augmentation pressure – a measure of reflected pressure wave, taken as the difference between maximum central systolic pressure and the pressure at first peak (Figure 1B); and Augmentation index – taken as the percentage of augmentation pressure to pulse pressure (AP/PP x 100).

### Definitions and Echocardiographic Measurements

Classification of AF was in accordance with expert consensus statement [13]. Paroxysmal AF was defined as recurrent AF that terminates spontaneously within 7 days. Persistent AF was defined as sustained AF lasting more than 7 days or between 2 and 7 days but requiring cardioversion. All echocardiographic measures were performed according to the American Society of Echocardiography guidelines [14]. In brief, left atrial measurements were taken using M-mode in the standard parasternal long axis view; and in 2-D apical 2- and 4-chamber views for measurements of left atrial area. Left atrial volume was calculated using the bi-plane area-length method and indexed for body surface area to derive left atrial volume index. Inter-ventricular septum was measured using both M-mode and 2-D from the parasternal long axis window. Left ventricular ejection fraction was calculated using the modified Simpson’s rule.

### Electrophysiology Study and Ablation

Electrophysiological procedures were performed with patients in the fasting state and under conscious sedation using midazolam and fentanyl. The ablation technique utilized at our institution has been previously described [8]. In brief, a single trans-septal puncture was performed under fluoroscopic guidance for left atrial access (BRK-1 needle, St Jude Medical, St Paul, MN, USA). The following catheters were used: Decapolar in the coronary sinus (Live wire Steerable, St Jude Medical); Circular for pulmonary vein mapping (Lasso Variable, Biosense-Webster, Diamond Bar, CA, USA); and 3.5 mm tip externally irrigated for ablation (Navistar or Celsius Thermocool, Biosense-Webster). Electroanatomic mapping system (CARTO, Biosense-Webster or Ensite NavX, St. Jude Medical) was used routinely for non-fluoroscopic navigation. Unfractionated heparin was administered in repeated boluses to maintain an activated clotting time of 300-350 seconds.

The ablation strategy included wide-encircling pulmonary vein ablation with an endpoint of pulmonary vein isolation in all patients. Further substrate modification was performed for patients with AF episodes lasting longer than 48 hours. This included linear ablation at the left atrial roof with endpoint of conduction block and/or electrogram-guided ablation at highly fractionated sites with abolition of fractionated signals as endpoint. Typically, radiofrequency power of 30W was used for pulmonary vein ablation and up to 35W for substrate modification while irrigation rate was fixed at 30 ml/min.

### Follow-Up

Patients were reviewed 3 monthly for the first year after ablation and 6 monthly thereafter. At each review, AF recurrence was ascertained from ambulatory 7-day Holter monitoring, 12-lead ECG and patients’ symptoms. All patients were treated with flecainide or sotalol for the first 6 weeks following the ablation procedure. Ongoing anti-arrhythmic drug use was at the discretion of the treating physician. Warfarin anti-coagulation was routine in all patients for a period of 3 months following ablation and continued in those with a CHADS_2_ score ≥2. Procedural success was determined as the absence of any atrial arrhythmia longer than 30 seconds without anti-arrhythmic drug use after a blanking period of 6 weeks.

### Statistical Analysis

Continuous variables were expressed as means ±SD or median with inter-quartile range accordingly using the Shapiro-Wilks test of normality. Data were compared using the unpaired Student’s t-test or Wilcoxon rank-sum test as appropriate. Categorical data were expressed as numbers and percentages, and compared using Fisher’s exact test. Kaplan-Meier survival analysis with log-rank testing was applied to compare post ablation AF recurrence. Statistical significance was established with two-tailed P value less than 0.05.

## Results

### Baseline Characteristics

Patient characteristics are presented in Table 1. Of the 68 lone AF patients, 43 had paroxysmal AF and 25 had persistent AF. Body mass index was similar between the two groups. Lone AF patients had significantly larger left atrial dimensions even though these were still within reference range for normal adults.

**Table 1:** Baseline Characteristics.

	**Control (n=50)**	**Lone AF (n=68)**	**P**
Age (yr)	**58.6±11.3**	**59.3±10.5**	**0.6**
Male sex (%)	**74.0**	**73.5**	**1.0**
Body mass index (kg/m^2^)	**26.9±4.4**	**27.5±4.5**	**0.5**
Duration of AF (months)	**-**	**60 (IQR 43-120**)	**-**
Longest AF episodes (days)	**-**	**2 (IQR 0.5-30**)	**-**
**Echocardiography**			
Left atrial parasternal size (cm)	**3.4±0.5**	**3.9±0.7**	**0.004**
Left atrial volume (ml)	**52.7±15.5**	**66.0±23.1**	**0.002**
Left atrial volume index (ml/m^2^)	**26.8±6.8**	**32.3±10.8**	**0.005**
Left atrial area (cm2)	**18.5±3.5**	**21.6±5.0**	**<0.001**
Inter-ventricular septum (mm)	**9.8±1.5**	**10.1±1.5**	**0.4**
Left ventricular ejection fraction (%)	**64±5**	**64±7**	**0.7**

IQR: Inter-quartile range.

### Peripheral and Central Pressures

**Figure 2:**
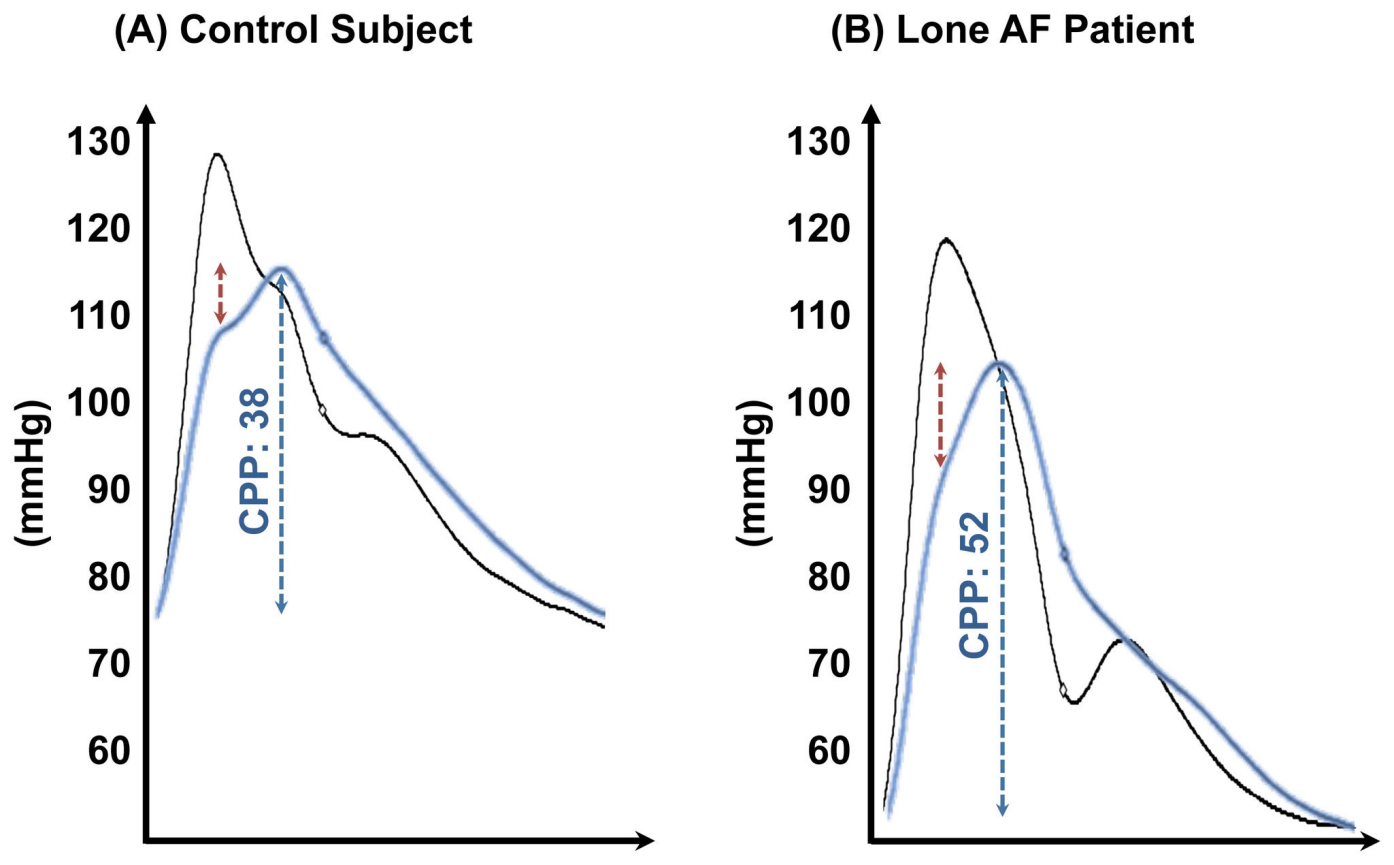
Examples of Peripheral and Central Pulse Waveforms. (A) Waveforms from a control subject showing peripheral pressures (black) of 128/76 with a pulse pressure of 52 mmHg and central pressures of 115/77 (blue) with a pulse pressure of 38 mmHg. Central augmentation pressure (red, *dotted*) is 7 mmHg. (B) Waveforms from a patient with lone AF showing peripheral pressures (black) of 120/54 with a pulse pressure of 66 mmHg and central pressures of 106/54 (blue) with a pulse pressure of 52 mmHg. Central augmentation pressure (red, *dotted*) is 12 mmHg.

The various peripheral and central blood pressure parameters are detailed in Table 2. Specifically, there was no significant difference in either peripheral or central systolic, diastolic & mean blood pressures. However, lone AF patients had significantly elevated measures of aortic stiffness: peripheral pulse pressure, central pulse pressure and augmentation pressure. Examples of peripheral and central pulse waveforms of the two groups are shown in Figure 2.

**Table 2:** Peripheral and Central Blood Pressures.

	**Control (n=50)**	**Lone AF (n=68)**	**P**
**Peripheral Pressures (mmHg)**			
Systolic	125±8	129±14	0.06
Diastolic	80±7	79±10	0.7
Mean	95±6	95±10	0.9
Pulse Pressure	45±8	50±12	0.009
**Central Pressures (mmHg)**			
Systolic	114±8	117±14	0.08
Diastolic	81±7	79±10	0.4
Mean	95±7	95±11	0.7
Pulse Pressure	33±6	38±11	0.02
Augmentation Pressure	6±3	9±7	0.04
Augmentation index (%)	21±10	22±13	0.5

### Aortic Stiffness and Atrial Fibrillation Recurrence

During a mean (±SD) follow-up of 2.9±1.4 years, 38 of the 68 lone AF patients had recurrence of AF at 31±27 weeks after single ablation procedure. As shown in Table 3, there was no significant difference in patient characteristics, AF type and echocardiographic parameters between cases and controls. Specifically, there was no difference in anti-hypertensive medication use including beta-blockers, renin-angiotensin system inhibitors, calcium channel blockers and diuretic agents.

**Table 3:** Characteristics and Hemodynamics by Atrial Fibrillation Recurrence.

	**No AF Recurrence (n=38)**	**AF Recurrence (n=30)**	**P**
Age (yr)	59.1±11.9	59.6±9.4	0.8
Male sex (%)	76.7	71.1	0.8
Body Mass Index (kg/m^2^)	27.2±4.2	27.7±4.8	0.7
Paroxysmal/Persistent AF (%)	63.3/36.7	63.2/36.8	1.0
**Echocardiography**			
Left atrial parasternal size (cm)	3.8±0.5	3.9±0.8	0.7
Left atrial volume (ml)	62.5±17.5	68.9±27.0	0.4
Left atrial volume index (ml/m^2^)	30.3±8.5	34.2±12.4	0.2
Left atrial area (cm^2^)	20.8±4.1	22.3±5.6	0.3
Inter-ventricular septum (mm)	10.1±1.9	10.9±1.7	0.09
Left ventricular ejection fraction (%)	65.2±7.4	63.5±7.0	0.3
**Peripheral Pressures (mmHg)**			
Systolic	127±14	130±14	0.3
Diastolic	80±10	78±10	0.8
Mean	95±10	95±10	1.0
Pulse Pressure	47±9	52±14	0.3
**Central Pressures (mmHg)**			
Systolic	116±13	119±14	0.4
Diastolic	81±10	78±10	0.4
Mean	95±11	95±11	1.0
Pulse Pressure	35±8	40±13	0.3
Augmentation Pressure	7±4	10±8	0.2
Augmentation index (%)	21±10	23±14	0.6

**Figure 3:**
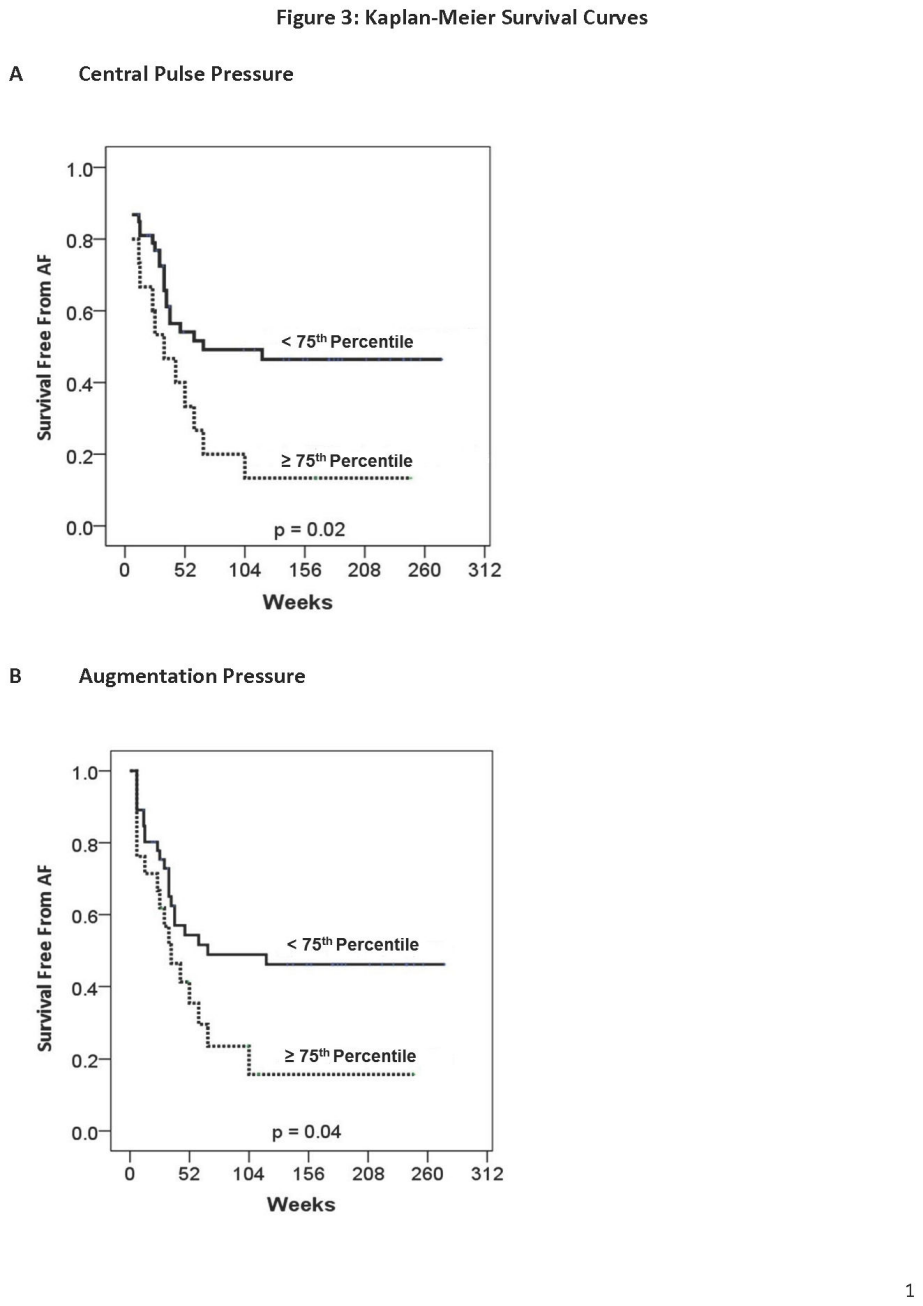
Kaplan-Meier Survival Curves. (A) Lone AF patients with central pulse pressure ≥75^th^ percentile had lower survival free from AF following catheter ablation. (B) Similarly, lone AF patients with augmentation pressure ≥75^th^ percentile had poorer outcome following catheter ablation.

Even though no differences were seen in both mean peripheral and central pressures, patients with highest levels of aortic stiffness (≥75^th^ percentile) had higher AF recurrence rates when compared to those with values less than the 75^th^ percentile (Peripheral pulse pressure ≥57mmHg: 82% vs. 47%, P=0.01; Central pulse pressure ≥45mmHg: 87% vs. 47%, P=0.008; Augmentation pressure ≥12mmHg: 76% vs. 48%, P=0.04; Augmentation index ≥33%: 76% vs. 49%, P=0.055). Importantly, corresponding analysis of those with highest levels of central or peripheral systolic, diastolic and mean blood pressure (≥75^th^ percentile) showed no relationship to higher AF recurrence rates. Likewise, left atrial dimensions (parasternal area & volume ≥75^th^ percentile) and inter-ventricular septal thickness (≥75^th^ percentile) were not significantly associated with higher AF recurrence (all P>0.7). In addition, only central aortic stiffness indices were associated with lower survival free from AF using Kaplan-Meier statistics (Central pulse pressure ≥75^th^ percentile, P=0.02 – Figure 3A; Augmentation pressure ≥75^th^ percentile, P=0.04 – Figure 3B; Augmentation index ≥75^th^ percentile, P=0.053 & Peripheral pulse pressure ≥75^th^ percentile, P=0.06 – both not illustrated).

## Discussion

### Major Findings

This study utilized well-validated, non-invasive central pulse wave analysis technique to examine central pressures and aortic stiffness in patients with lone AF who had no apparent structural heart disease and risk factors for AF. The major findings are as follows:

1 Increased aortic stiffness (higher pulse pressure and augmentation pressure) was evident in lone AF patients as compared to controls;
2 Measures of aortic stiffness demonstrated stronger association with AF recurrence following catheter ablation than standard brachial blood pressure measures and conventional echocardiographic parameters;
3 Lone AF patients had larger left atrial dimensions than controls.

Therefore, this study implicates aortic stiffness as a silent contributing factor in the development of lone AF. Elevated aortic stiffness, by leading to abnormal atrial remodeling, creates a path to development of the substrate that develops apparently “lone” AF.

### Aortic Stiffness: A Silent Factor Leading to Remodeling and Atrial Fibrillation

Studies in lone AF patients have alluded to several underlying atrial abnormalities thereby pointing to the possibility of as yet unrecognized risk factors. These abnormalities include: increased inflammation, diastolic dysfunction, increased fibrosis and microvascular dysfunction [15-18]. More recently, our group has demonstrated further evidence of significant electrical and structural changes in lone AF atria [8,9]. This included structural abnormalities evidenced by loss of myocardial voltage, conduction slowing, sinus node dysfunction and prolonged atrial refractoriness. Non-invasive delayed enhancement magnetic resonance imaging has also demonstrated this abnormal atrial substrate [10]. As such, lone AF patients are ideal candidates for studies aiming to uncover novel AF risk factors in the absence of structural heart disease [4,5]. As seen in this study, both peripheral and central measures of aortic stiffness were higher in lone AF patients and associated with higher arrhythmia recurrence following catheter ablation. Aortic stiffness may signify an ‘atrial-myopathy’ in patients who have apparent normal left atrial dimensions, ventricular thickness and brachial systolic blood pressure. The present study also affirms the findings of the Framingham study linking aortic stiffness to the development of AF [2]. We believe aortic stiffness is an unrecognized silent factor in lone AF contributing to occult abnormal atrial remodeling.

### Causes of Aortic Stiffness: Implications in Atrial Fibrillation

The causes of aortic stiffness remain incompletely understood. The lack of association between aortic stiffness and conventional cardiac risk factors other than hypertension and aging has been well documented [19-21]. Other possible contributing factors include inflammation and mechanical stress [20,22,23]. Interestingly, recent reports had also found increased arterial stiffness and P-wave duration in subjects with pre-hypertension [24,25]. Indeed, systolic blood pressure in the pre-hypertensive range has been shown to be independently associated with incident AF in a large prospective study [26]. Furthermore, pre-clinical work has shown a relationship between short-term hypertension and increased atrial inflammation leading to a substrate for AF [27]. The atrial remodeling process in the same hypertensive model was progressive, highlighting the potential benefits of early treatment to prevent formation of an arrhythmogenic substrate [28]. Taken together, aortic stiffness may identify a subset of at-risk patients in the pre-hypertensive spectrum. Recognition and targeting of aortic stiffness in patients with or at risk of AF may be important in our bid to reduce the burden of this emerging epidemic.

### Aortic Stiffness: Central versus Peripheral Measures

Theoretically, central pressure is more patho-physiologically relevant to the heart than their peripheral counterparts as its proximity will exert greater strain leading to increased afterload and impaired relaxation [5]. A recent systematic review and meta-analysis highlighted the value of central hemodynamic indices in predicting cardiovascular outcomes [3]. Central pulse wave analysis from applanation tonometry of the radial artery has been accepted as a valid measure of subclinical target organ damage. Central estimates of aortic stiffness as used in this study provided additional information that may also be useful for monitoring of therapeutic effects. Recently, the CAFÉ investigators have shown that different anti-hypertensive agents can have significantly different effects on central aortic pressures despite similar brachial readings [29]. Specifically, amlodipine±perindopril combination had greater effects on central pressures than atenolol±thiazide. While heart rate reduction with beta-blockade is thought to be responsible for the less effective central pressure reduction, whether the difference in central aortic pressures could explain the different clinical outcomes in these 2 groups remains unknown [30]. Nevertheless, further evaluation is required to assess central aortic stiffness in AF.

### Clinical Implications

This study affirms aortic stiffness as a novel modifiable risk factor for AF. It can be used to identify “high-risk” pre-hypertensive patients with subclinical atrio-myopathy. Further studies are necessary to determine whether targeting of aortic stiffness with anti-hypertensive medications may modify the burden or progression of AF. Of note, agents with central blood pressure effects such as inhibitors of the renin angiotensin system have also been shown to reduce the occurrence of AF in various patient groups including normotensive lone AF patients [31,32].

### Study Limitations

We did not have longitudinal measures of central pulse wave analysis. Inclusion of pulse wave velocity measurements would have strengthened our findings. Pulse wave analysis ideally requires stable artery waveforms, which is difficult when a subject is in AF.

## Conclusions

Lone AF is associated with greater aortic stiffness; which contributes to higher AF recurrence following catheter ablation procedures. Active monitoring and targeting of this novel risk factor may improve outcomes in patients with AF.
